# A Sixty-Hour Week for Mental Hospital Workers

**Published:** 1912-05-25

**Authors:** 


### The Hospital

The Workers' Newspaper of Administrative Medicine and Institutional Life, Administration, National Insurance and Health.

No. 1350, Vol. LII. SATURDAY, MAY 25, 1912. PRICE ONE PENNY.

### A SIXTY-HOUR WEEK FOR MENTAL HOSPITAL WORKERS

A determination on the part of organised labour to claim not only the necessaries of life but also opportunities for the enjoyment of some of its legitimate pleasures is a characteristic of our time. Our system of education creates tastes which cannot be gratified under existing conditions, while giving the worker a keener conception of the limitations of his lot. That the leaven of unrest is working among those who spend their days in the care of mental patients is not surprising to us who are familiar with the subject. Shorter hours on duty and more leisure are being demanded by the mental attendant and nurse. It is impossible for an outsider to judge the merits of this claim or to say whether a maximum of 60 hours on duty per week in a mental hospital ward is a fair week's work. When, however, a Commissioner in Lunacy, in giving evidence before a Select Committee of the House of Commons, states that the average number of hours during which attendants in our mental hospitals are on duty is 84, even laymen will not doubt that the request for some reduction is reasonable. We are only surprised that it has not been advanced before and that it is not more insistent now. It is true that the work of all those employed in mental hospitals is not equally trying. In some cases it approximates to that of domestic service, but even at its best it necessitates constant association with the insane.

On the other hand, the nursing of the acutely insane and of many of the chronic insane and idiotic is as trying and responsible an occupation as any man or woman can undertake. It is not free from the risk of bodily injury, while it entails the performance of duties which the general public would, and with reason, describe as disgusting. There can be no doubt that an average of 84 hours per week is too much for any man or woman to spend in work of this class. Some mental hospitals, amongst which those under the control of the London County Council deserve honourable mention, do not ask such long hours from their staffs. It is not creditable that those working in institutions under the control of county and borough councils should find it necessary to appeal to Parliament to enact that there shall be a statutory limitation of these hours of duty. The idea of limiting by law the length of time which an attendant or nurse shall be in charge of his or her patient is not one which in itself appeals to us, and we can easily conceive circumstances which might render such an enactment both harmful and difficult to carry out. If, however, it be the only method of bringing public bodies to a correct sense of what is due to men and women who perform a duty necessary to the community, and one which is difficult, distasteful, and even dangerous, we regretfully welcome the compulsion. The Parliamentary Committee of the Medico-Psychological Association accept this principle of statutory limitation of hours, though the Scottish Division of the same Association somewhat illogically expressed their strong disapproval. They proposed that in its place time-tables showing the working hours should be prepared for each institution and be subject to the sanction of the General Board of Lunacy. In effect this would amount to an official limitation of hours. The original proposal in the Asylums Officers (Employment, Pension, and Superannuation) Bill, which was sent before a Select Committee, was that there should be a statutory maximum of 60 hours weekly. To this there was strong opposition. It was urged that it would entail a three-shift system for the 24 hours, and, in the opinion of those best qualified to judge, it was held that under such a system the patients would suffer from the more frequent change of their attendants. There was also strong opposition on the ground of expense. It was calculated that the additional staff that would be required for England and Wales if this alteration were made would cost £600,000 annually, and that in addition to this it would be necessary to provide buildings for their accommodation. The representatives of the staffs were not themselves unanimous in their support of a 60-hours week. The Select Committee took a wise course in recommending that a maximum of 70 hours for day duty and 60 hours for night duty should not be exceeded, and, in order to overcome the difficulties in administration which were recognised, a fortnightly period instead of a weekly one was decided upon. The hours on duty per fortnight must not exceed 140 for day duty and 120 for night duty. While fixing this maximum the Committee express the opinion that authorities in charge of mental hospitals might with advantage consider whether further reductions could not be made. We would strongly urge upon those who are responsible for the management of those institutions in which the nursing staff is on duty for such long hours to reduce these hours as an act of grace, and not wait until it is wrung from them by Act of Parliament. The time is not ripe for a general reduction to 60 hours per week, even if it were altogether desirable. We would prefer a system by which the senior attendants who are in charge, and on whom the burden of responsibility lies, should have shorter hours than the junior members and those whose work is less trying. For the former 60 hours may be quite enough; the latter may well be called upon to do 70.

